# Antioxidant capacity of *Pleurotus ostreatus* (Jacq.) P. Kumm influenced by growth substrates

**DOI:** 10.1186/s13568-024-01698-0

**Published:** 2024-06-15

**Authors:** Hailu Gebru, Gezahegn Faye, Tolosa Belete

**Affiliations:** 1 Department of Horticulture, College of Agriculture and Natural Resources, Salale University, P.O. Box 245, Fiche, Ethiopia; 2 Department of Chemistry, College of Natural Science, Salale University, P.O. Box 245, Fiche, Ethiopia; 3 Department of Biology, College of Natural Science, Salale University, P.O. Box 245, Fiche, Ethiopia

**Keywords:** Antioxidant capacity, Functional constituents, Mixture substrate, RSA, Vitamin C, Vitamin D

## Abstract

Functional constituents are the main concern in food production and consumption. Because foods rich in functional constituents have antioxidant capacity and are important in keeping consumers healthy. *Pleurotus ostreatus* is among foods rich in functional constituents. However, its functional constituents are affected by various factors. This study compared the antioxidant capacity of *P. ostreatus* grown on different substrates: straws of *tef* (Trt1), barley (Trt2), and wheat (Trt3), husks of faba bean (Trt4), and field pea (Trt5), sawdust (Trt6), and the mixture of the above with 1:1 w/w (Trt7). Trt7 had significantly higher radical scavenging activity (RSA) (73.27%), vitamin C (10.61 mg/100 g), and vitamin D (4.92 mg/100 g) compared to other treatments. Whereas the lowest values of RSA (44.24%), vitamin C (5.39 mg/100 g), and vitamin D (1.21 mg/100 g) were found in Trt2. The results indicated that mixed substrate may be a good growth substrate for functionally beneficial *P. ostreatus* and could be a promising source of natural antioxidants.

## Introduction

Oyster mushroom, *Pleurotus ostreatus* (Jacq.) P. Kumm is a rich source of functional constituents (Dicks and Ellinger 2020). The functional constituents have antioxidant capacity and can protect living cells from death related to aging (Liuzzi et al. 2023) which commonly occur as by-products of normal physiological processes (Warraich et al. 2020). Mushrooms have been selected as one of the most popular food ingredients in the human diet due to their nutritional (Kalač 2009), medicinal (Jędrejko et al. 2021), unique taste and flavour (Fernandes et al. 2013), and aroma (Ng et al. 2022). Recently, mushroom production has been getting special attention in Ethiopia (Zeleke et al. 2020). The common edible mushrooms, which are mostly grown not only in Ethiopia but also in the world include *Pleurotus ostreatus* (Jacq.) P. Kumm, *Agaricus bisporus* (J.E. Lange) Imbach, and *Lentinula edodes* (Berk.) Pegler (Thakur 2020).

Edible mushrooms contain various nutritional compounds such as proteins (Assemie and Abaya 2022), carbohydrates (El-Maradny et al. 2021), vitamins (Marçal et al. 2021), dietary fibers (Wang et al. 2021), and low content of fats (Cheung 2010). More importantly, they also produce a variety of secondary metabolites (Fukushima-Sakuno 2020) such as numerous alkaloids (Sakamoto et al. 2020), terpenes (Al-Salihi and Alberti 2021), steroids (Baosong et al. 2020) and phenolic compounds (Abdelshafy et al. 2022). The secondary metabolites which have antioxidant capacity possess anti-microbial activity (Meshram et al. 2013), anti-genotoxic (Martins De Oliveira et al., 2002), anti-oxidant (Shaffique et al. 2021), anti-proliferative (Yap et al. 2013), anti-cancer (Patel and Goyal 2012), anti-hyperlipidaemia (Ren et al. 2017), anti-nociceptive (Barahona et al. 2012), immunestimulanting (El Enshasy and Hatti-Kaul 2013), hypocholesterolaemia (Abidin et al. 2017), antiatherogenic (Rauf et al. 2023), anti-allergic (Jayachandran et al. 2017), and neuroprotective (Rai et al. 2021). Thus, they can be used for therapeutic purposes (Chaturvedi et al. 2018). Such antioxidants have better radical scavenging properties (Yap et al. 2015) and able to protect against oxidative destruction of biomolecules (lipids, proteins, DNA) (Mwangi et al. 2022) ultimately leading to many chronic diseases such as cancer (Thanan et al. 2015), cardiovascular diseases (Roychoudhury et al. 2021) and inflammation in humans (Mehdi et al. 2021).

Constituents that make mushrooms significantly functional foods (Martinez-Medina et al. 2021) are a diverse group of phytochemicals or bioactive compounds (Shibamoto et al. 2008) that can have beneficial effects on human health (Thu et al. 2020). So, they have been used in folk medicine throughout the world since ancient times (Bita et al. 2022). Mushrooms contain a large number of biologically active components that offer health benefits and protection against many degenerative diseases (Nitha et al. 2010). Several medicinal mushrooms have been reported to possess significant antioxidant activity (Alvarez-Parrilla et al. 2007). Some of the isolated and identified substances from mushrooms have been reported to possess significant anticancer, cardiovascular, antiviral, antibacterial, anti-parasitic, hepatoprotective, and antidiabetic activities (Ooi and Liu 2000).

Not only their proper growth and yield but also the amounts of nutritional and functional constituents contained in mushrooms are the result of internal (genetic) factors, external (environmental) factors, and the interaction effect of the two factors. The production of active ingredients in medicinal plants is guided by genetic processes, and it is also strongly influenced by environmental factors (Yuan et al. 2020). Therefore, environmental factors cause changes in the growth of medicinal plants, as well as the quantity and quality of their active ingredients, such as alkaloids, glycosides, steroids, and essential oils (Pant et al. 2021). Growth substrates are among the externally (environmental) affecting factors of the functional constituents of the mushrooms (Koutrotsios et al. 2022).

The antioxidant capacity of *P. ostreatus* is thus affected by the type of substrate on which the mushrooms are grown. Of course, many external (environmental) factors such as water, air, soil, elevation, differences between species, extraction methods, and antioxidant measurements affect the number of secondary metabolites in plants, including phenol and flavonoids (Zargoosh et al. 2019). Although Nitha et al. (2010), Jayakumar et al. (2011), Roupas et al. (2012), Manninen et al. (2018), Bahadori et al. (2019), Moumita and Das (2022), and Mwangi et al. (2022) are among many researchers who have been investigated on the general antioxidant effects of mushrooms so far, the reports on the influence of growth substrates on the antioxidant capacity of oyster mushrooms are still lacking. Therefore, this study aimed to investigate the influence of growth substrates, which are selected based on their economic accessibility and environmentally friendly, on the antioxidant capacity of *P. ostreatus*.

## Materials and methods

### Substrate preparation

The crop by-products (straws of *tef*, barley, & wheat, and husks of faba bean & field pea) were collected from the nearby peasant associations of Girara-Jarso district and the sawdust was obtained from a woodwork shop at Fiche town, and prepared following methods indicated by Thuc et al. (2020). Accordingly, the collected substrates were cleaned with tap water, air dried and chopped into pieces of about 3–5 cm in size. Then, they were naturally dried by exposure to full sun for three days. Consequently, the substrates were soaked in water overnight and then sterilized by hot water under the temperature range of 70–80 °C for 30 min. The substrates were then spread on the clean plastic-covered floor for evaporation of excess moisture; and when the water stopped dripping, they were considered ready for spawning.

### Treatments and experimental design

Treatments consisted of seven lignocellulose substrates such as straws of *tef* [*Eragrostic tef* (Zucc.) Trotter] (TS = Trt1), barley (*Hordeum vulgare* L.) (BS = Trt2), & wheat (*Triticum* sp. L.) (WS = Trt3), husks of faba bean (*Vicia faba* L.) (FBH = Trt4) & field pea (*Pisum sativum* L.) (FPH = Trt5), sawdust (SD = Trt6), and the mixture of them (1:1 ratio w/w) (MIX = Trt7) (Table 1) were laid out in a completely randomized design (CRD) with three replications per treatment.

**Table 1:** Treatments and substrate composition for *P. ostreatus* cultivation

Treatments	Substrate composition	Moist weight per bag
Trt1	TS (100%)	1 kg
Trt2	BS (100%)	1 kg
Trt3	WS (100%)	1 kg
Trt4	FBH (100%)	1 kg
Trt5	FPH (100%)	1 kg
Trt6	SD (100%)	1 kg
Trt7	TS + BS + WS + FBH + FPH + SD = MIX	0.167 kg each*6 = 1 kg

### Spawning of Pleurotus *ostreatus*

The pure culture of the *P. ostreatus*, strain M2153 was obtained from Waginos Biotech Mushroom Spawn Production PLC (Addis Ababa, Ethiopia). The mushroom, *P. ostreatus* was selected based on data from previous studies (Valverde et al. 2015). It is characterized by its adaptation to tropical and subtropical environment naturally and can be artificially cultivated due to its ability to colonize and degrade a wide variety of substrates containing cellulose, hemicellulose, and lignin, using them in its development. Furthermore, it has quick mycelium growth and fruiting and a low cost of culture, being slightly affected by diseases, and requiring minimal monitoring of the cultivation environment due to easy adaptation and maintenance. Therefore, and also due to nutritional and functional characteristics, *P. ostreatus* is considered increasingly popular from a commercial point of view.

One kg of each of the moist substrates was spawned with 70-gram seeds of the *P. ostreatus* in transparent plastic bags of 43 cm by 30 cm dimensions following processes indicated by Chang and Miles (2004). Accordingly, ten holes were made in each bag for adequate aeration and the plastic bags were tied and incubated in the dark and well-ventilated cropping room. After spawning, the bags were kept about 20 cm apart in the cropping room in temperature and relative humidity ranges of 25–30ºC and 80–90%, respectively. Fruiting was started shortly after the residue was filled with mycelia growth. Sisal-made sacks were side-hanged, and water was sprinkled on the hanged sack and plastic-covered floor twice a day to maintain high humidity in the cropping room. The mushrooms were harvested from the substrates when young, firm, and fleshy (immature/juvenile stage). Harvesting was performed by gently pulling the mushrooms from the residues and continued as long as the mycelia remained white and firm and a total of three flushes were harvested.

### Procedures for extracting functional constituents

Since dried samples gave higher 2, 2-diphenyl-1-picrylhydrazyl (DPPH) scavenging activity (Fernandes et al. 2013), oyster mushroom samples were powdered with an electric blender after drying. Maceration method of extraction was used in which 2 g of each of the powdered samples were added to 40 mL of methanol (80%) in a round bottom flask and the extraction was processed for about 7 h with shaking using KS Oscillator. The extract was filtered with Whitman filter paper and kept in the refrigerator until used for antioxidant activity experiments.

### Determining the functional constituents

The antioxidant activity and IC_50_ values of the samples were determined by mixing mushroom extracts with DPPH solution in methanol by using a slight modified method of Baliyan et al. (2022). Four mL of DPPH solution (3 × 10^− 4^ mg/mL) were mixed in test tubes with 4 mL extract solutions of different concentrations (5 mg/mL, 10 mg/mL, 15 mg/mL), kept for 30 min of incubation period at dark place, and absorbance was measured at 517 nm. Then, the RSA (%) was determined by using the formula of Baliyan et al. (2022) mentioned below.

RSA (%) = [(Ac-As)]/Ac x100.

*Where, As = the absorbance of the sample and Ac = the absorbance of the negative control (DPPH solution).*

To determine the influence of growth substrates on the antioxidant capacity of the *P. ostreatus*, different analytical methods were followed and their results were expressed on a dry weight basis to record the appropriate comparison between them. The vitamin C (mg/100 g) content of the mushroom was determined by the spectrophotometer described by the Nielsen (2010) standard method whereas the content of vitamin D was determined following (AOAC 1990) procedures.

### Statistical analysis

The collected data were subjected to analysis of variance (one way) using the software of Statistix 8.0. Significances among treatment means were tested using LSD test *P* < 0.05 (Gomez and Gomez 1984). To show the relationships between the variables under study, a linear regression analysis was run based on the antioxidant compositions of *P. ostreatus*.

## Results

### Radical scavenging activity and IC_50_ values of *P. ostreatus* influenced by growth substrates

**Fig. 1:**
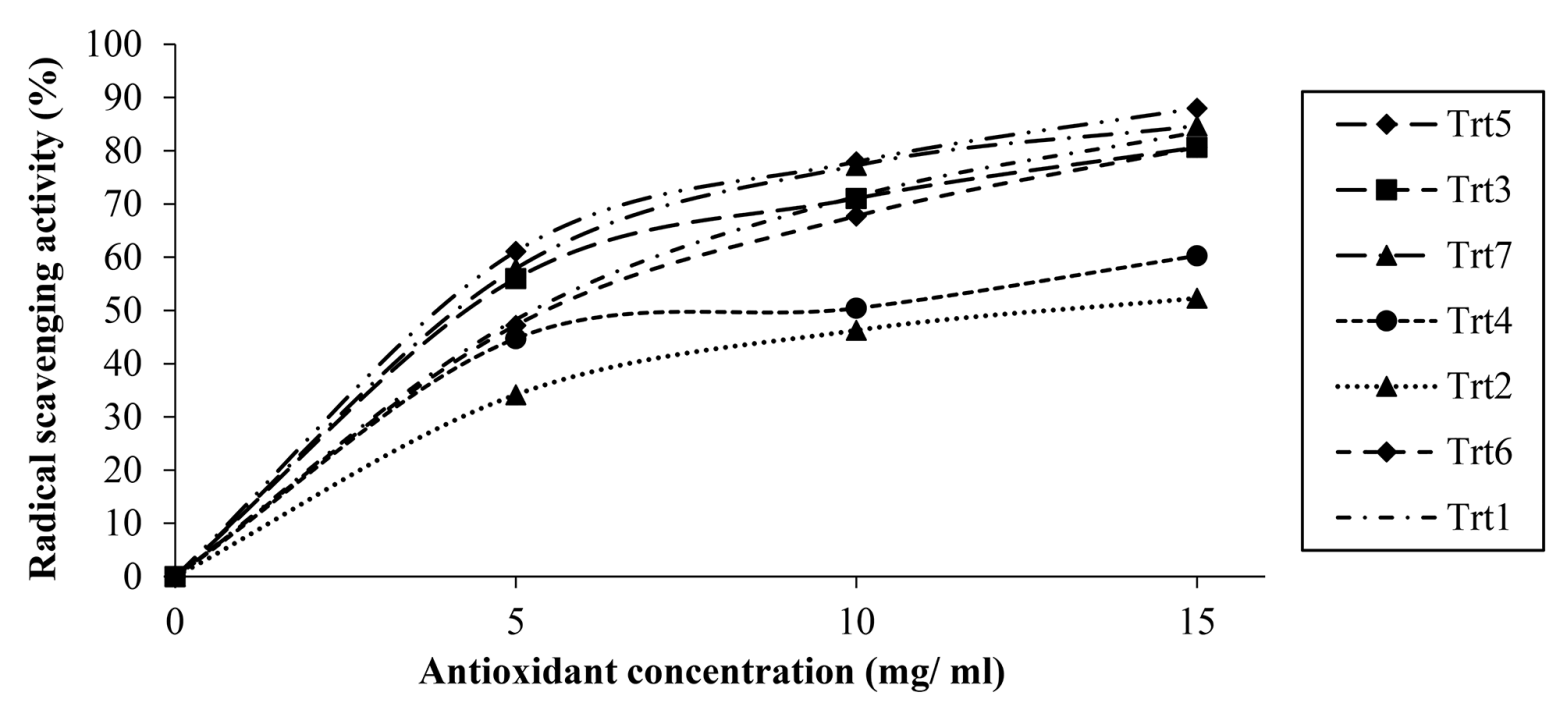
Radical scavenging activities of *P*. *ostreatus* influenced by the antioxidant concentration

The RSA of the *P. ostreatus* cultivated on various substrates varied from 44.24 to 75.68% (Table 2) and increased with an increase in concentration (Fig. 1). It was higher for Trt6, Trt1, Trt3, Tert7, and Trt5 (65.20-75.68% at 15% concentration) in comparison to Trt2, the lowest (44.24%) RSA. The RSA results were consistent with the vitamin C contents of *P. ostreatus.* The higher the vitamin C content in *P. ostreatus* cultivated on Trt5 and Trt7 substrates, the better the RSA of the mushroom (Table 2).

**Table 2:** Radical scavenging activity and IC_50_ values of *P. ostreatus* influenced by growth substrates

Parameter	Treatments						
	Trt1	Trt2	Trt3	Trt4	Trt5	Trt6	Trt7
RSA (%)	67.73^abc^	44.24^c^	69.24^ab^	51.80^bc^	75.68^a^	65.20^abc^	73.27^ab^
IC_50_ (mg/ml)	4.97^d^	13.17^a^	2.20^e^	8.84^b^	0.46^g^	5.45^c^	1.32^f^

Means with the different letters in the same row are significantly different (α < 0.05)

Thus, the *P. ostreatus* harvested from the Trt5 substrate showed the highest RSA (88.02%) at 15 ml/mL concentration; whereas, the *P. ostreatus* samples harvested from the Trt2 showed the least RSA (34.16%) at 5 ml/mL concentration. The IC_50_ values in the DPPH assay varied from 0.46 to 13.17 mg/ml for all samples tested (Table 2). It was observed that the IC_50_ values showed the reverse to the RSA; i.e., as RSA increases, the IC_50_ values decrease with the order of Trt2 > Trt4 > Trt6 > Trt1 > Trt3 > Trt7 > Trt5.

### Vitamin C contents of *P. ostreatus* influenced by growth substrates

The ascorbic acid (vitamin C) content ranged between 5.39 mg/100 g for Trt2 and 10.71 mg/100 g for Trt5 (Table 3). The vitamin C content (10.61 mg/100 g) obtained in the *P. ostreatus*, which grew on the Trt7 substrate was significantly (*P* < 0.05) comparable to that of the maximum one, which grew on the Trt5 substrate.

**Table 3:** Vitamin C & D contents of *P. ostreatus* influenced by growth substrates

Parameter	Treatments						
	Trt1	Trt2	Trt3	Trt4	Trt5	Trt6	Trt7
Vitamin C (mg/100 g)	9.36^c^	5.39^f^	8.24^d^	10.30^b^	10.71^a^	7.80^e^	10.61^a^
Vitamin D (mg/100 g)	1.54^b^	1.21^f^	1.30^e^	1.44^d^	0.97^g^	1.48^c^	4.92^a^

The different letters in the same row are significantly different (α < 0.05)

### Influence of growing substrates on vitamin D contents of *P. ostreatus*

In the present study, the value of vitamin D obtained ranged between 0.97 for Trt5 and 4.92 mg/100 g for Trt7 (Table 3). Compared to the other functional constituents, the amount of vitamin D obtained from the *P. ostreatus* grown on different substrates are minimum.

### Linear regression

**Fig. 2:**
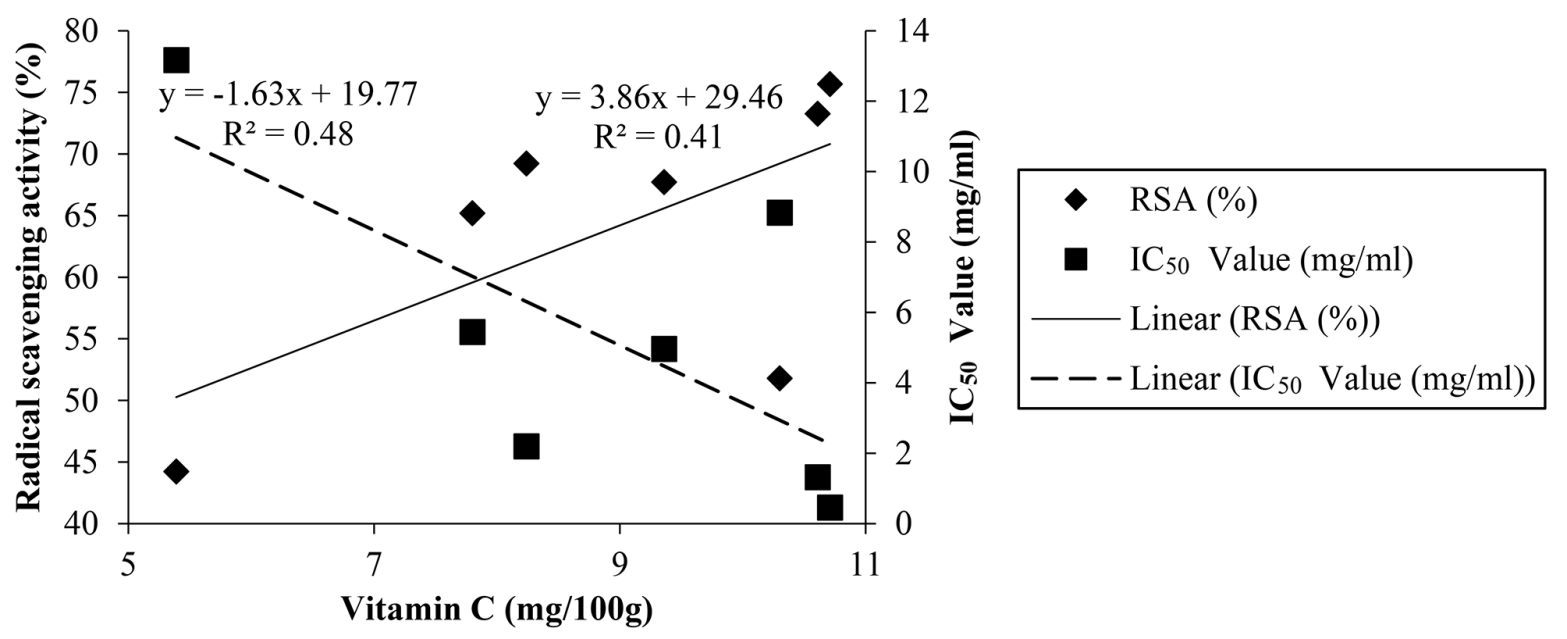
Linear relationships among radical scavenging activity (RSA %), vitamin C (mg/100gm), and IC_50_ Values (mg/100gm) of *P. ostreatus*

**Fig. 3:**
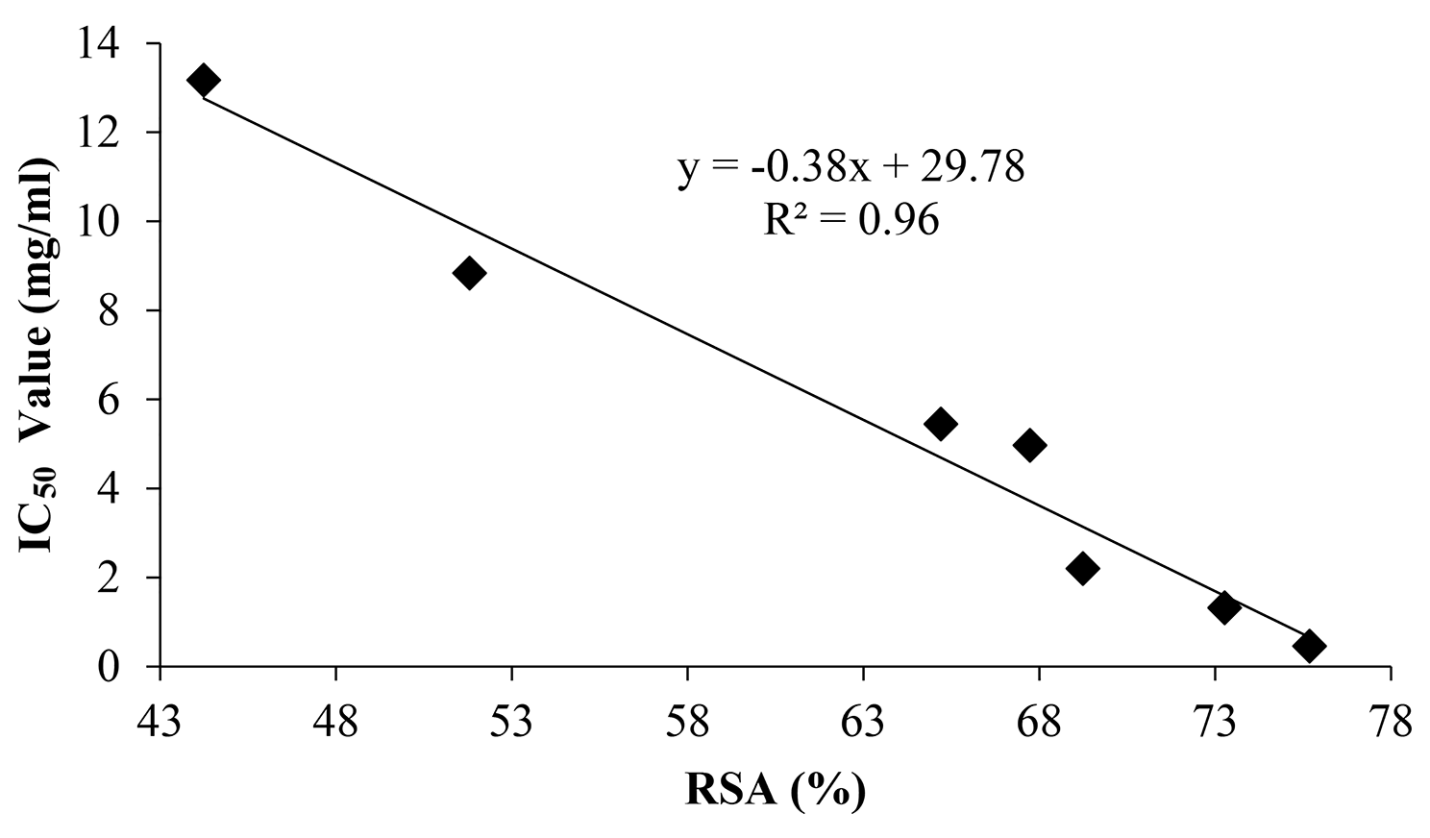
Linear relationship between RSA (%) and IC_50_ Value (mg/ml)

The results indicated that there were statistically significant (*P* < 0.05) relationships among vitamin C, radical scavenging activity, and the IC_50_ value (Figs. 2 and 3). Accordingly, the radical scavenging activity was found to have a positive and statistically significant relationship; while the IC_50_ value was found to have a negative and statistically significant relationship with vitamin C contents of the *P. ostreatus*.

## Discussion

### Radical scavenging activity and IC_50_ values of *P. ostreatus* influenced by growth substrates

Free radical scavenging is one of the methods of inhibiting lipid oxidation commonly used to estimate antioxidant activity. In the present study, the RSA of *P. ostreatus* extracts was tested against the DPPH and higher for the Trt7 and Trt5. The study results further indicated that as the concentration of antioxidants increased from 5 ml/mL to 15 ml/mL, the RSA (%) of the mushroom extract also increased from 49.91 to 75.73% on average. Similarly, Nitha et al. (2010) and (González-Palma et al. 2016) reported that the RSA was increased with increasing concentrations of the antioxidants.

On the other hand, the IC_50_ value of the sample extracts was the reverse. That means the IC_50_ value decreased as the concentration of antioxidants increased. The results found in the present study are similar to the reported IC_50_ values of Bakir et al. (2018). Pumtes et al. (2016) also reported that the IC_50_ of a compound is inversely related to its antioxidant capacity, as it expresses the amount of antioxidants required to decrease the DPPH concentration by 50%. Furthermore, at a lower value of IC_50_, the extract had higher antioxidant activity (Samruan et al. 2012).

The extract using 80% methanol (v/v) was assumed to be the strongest inhibitor which showed IC_50_ of DPPH assay at the lowest concentration (0.46 mg/ml) among all sample extracts, whereas the weakest inhibitor that showed IC_50_ of DPPH assay at the highest concentration (13.17 mg/ml) significantly (*p* < 0.05). These results compared to the extract from *P. ferulae* (IC_50_: 4.55 mg/ml) (Tsai et al. 2009), P. *eryngii* (IC_50_: 8.67 mg/ml) (Reis et al. 2012), P. *pulmonarius* (IC_50_: 6.00 mg/ml) (Arbaayah and Umi Kalsom 2013), and *L. edodes* (IC_50_: 9.8 mg/ml) (Woldegiorgis et al. 2014) which have significant antioxidant activities.

### Vitamin C contents of *P. ostreatus* affected by growth substrates

Functional constituents such as vitamin C, which has natural antioxidant properties and can scavenge free radicals, are also investigated in *P. ostreatus* extracts and their amount was significantly (*P* < 0.05) influenced by the growth substrate types. The RSA results were consistent with the vitamin C of the *P. ostreatus*. The higher the vitamin C content the better RSA observed. The scavenging effects of the extracts from the fruiting bodies of *P. ostreatus* on DPPH radicals increased with increasing concentrations.

The amounts of vitamin C content obtained in this study ranged between 5.39 and 10.71 mg/100 g for Trt2 and Trt5, respectively, are far less when compared to the results of other researchers. For example, Patil et al. (2010) found 12.52 ± 0.3 to 15.80 ± 0.8 mg/ 100 g of vitamin C contents of *P. ostreatus* growing on different agro-wastes; while Zahid et al. (2020) obtained 32.1 to 44.8 mg/100 g contents of vitamin C in *P. ostreatus* from the control (pure cotton waste) and cotton waste amalgamated with 10 mM/L of humic acid, respectively; and concluded that oyster mushroom cultivated on cotton waste enriched with humic acid provided a favourable media for mushroom growth with a significant increase in vitamin C contents and other macro and micro nutrients.

### Influence of growth substrates on vitamin D contents of *P. ostreatus*

Vitamin D is important for calcium absorption and bone health, and its deficiency can lead to softening of the bone in children and adults as well as osteoporosis in adults (Roberts et al. 2008). The two general molecules of vitamin D are cholecalciferol (vitamin D_3_) and ergocalciferol, the direct precursor of vitamin D_2_ (Nowson et al. 2012). Vitamin D intake comes naturally from sunlight and a limited number of foods such as UV-B exposure of mushrooms (Ahlborn et al. 2019). Mushrooms can grow well in both outdoor and indoor conditions; however, outdoor cultivation has risks of exposure to rain, wind, and/or high temperatures, all of which reduce yield. Thus, the yield of indoor mushroom production is higher and more stable, as such, indoor growing is preferred (Thuc et al. 2020). However, some mushrooms that have been exposed to UV radiation provide 800 IU (20 g) of vitamin D_2_ per 100 g (Holick 2007). Ergosterol, located in the cell membrane of fungi, is easily transformed into vitamin D_2_ by UV-B exposure (Roberts et al. 2008). The low amount of vitamin D content (0.97 and 4.92 mg/100 g) in this study might be due to the non-exposure of the mushroom to sunlight. Roberts et al. (2008) and Ahlborn et al. (2019) reported that the maximum vitamin D_2_ concentration formed was 12.48 and 6.2 µg/g dry solids using 5.04 µg/h and 1.0 mW/cm^2^ UV-B exposure, respectively, in dry mushrooms.

### Linear regression

Linear relationships were observed among vitamin C, radical scavenging activity, and the IC_50_ value. Consequently, as the ingredient of vitamin C increased by 1 mg/100 g, the radical scavenging activity of *P. ostreatus* increased by 3.86%. This can be related to the fact that as vitamin C content increases in *P. ostreatus*, the radical scavenging activity of cells will increase due to the positive relationship between them. On the other hand, as the constituent of vitamin C increased in the *P. ostreatus* by 1 mg/100 g, its IC_50_ value will be decreased by 1.63 mg/mL due to the negative relationship between them. Similarly, as the IC_50_ value increased by 1 mg/mL, its radical scavenging activity decreased by 0.38% since they are negatively related. The results of Zargoosh et al. (2019) also confirm this linear correlation.

In general, among the substrates tested, the Trt7 produced a fruiting body with better antioxidant activity in terms of RSA on the DPPH free radical. Having higher amounts of vitamin C with the presence of radical scavenging activity, consumption of *P. ostreatus* growing on a mixture substrate (Trt7) might be beneficial to protect the human body against oxidative damage, which can further develop into health-related degenerative illnesses. Because, *P. ostreatus* exhibited antioxidant activities, which are influenced by growth substrates. This powerful protective effect of *P. ostreatus* suggests that it can serve as a promising source of natural antioxidants and growers can, therefore, use the mixture of the substrates instead of using single agriculture by-products to produce mushroom products with higher amounts of functional constituents. Further work is necessary to promote the development of value-added functional constituents from *P. ostreatus* for use in the food and pharmaceutical industry.

## Data Availability

There are no other data than the row data that are already used for the analysis.

## Abbreviations

Ac: Absorbance of the negative control
AOAC: Official Methods of Analysis of Association of Analytical Chemists
As: Absorbance of the sample
BS: Barely straw
CNS: Central nervous system
CRC: Chemical Rubber Company
CRD: Completely randomized design
DNA: Deoxyribonucleic acid
DOI: Digital object identifier
DPPH: 2,2-diphenyl-1-picrylhydrazyl
FBH: Faba bean husk
FPH: Field pea husk
IC_50_: Half maximal inhibitory concentration
IU: International units
KS: Kohn-Sham
LSD: Least significant difference
MIX: Mixture
PLC: Public limited company
RSA: Radical scavenging activity
SD: Sawdust
TS: *Tef* straw
USA: United States of America
UV: Ultraviolet
UV-B: Ultraviolet B
WS: Wheat straw
